# Effect of Biomimetic Fish Scale Texture on Reciprocating Friction Pairs on Interfacial Lubricating Oil Transport

**DOI:** 10.3390/biomimetics10040248

**Published:** 2025-04-17

**Authors:** Tao Sun, Zhijun Yan, Lixia Xue, Yuanyuan Jiang, Shibo Wu

**Affiliations:** 1 Marine Engineering College, Dalian Maritime University, Dalian 116026, China; suntao708@dlmu.edu.cn (T.S.); xlx2.2@dlmu.edu.cn (L.X.); 2 Transportation Engineering College, Dalian Maritime University, Dalian 116026, China; jyy@dlmu.edu.cn (Y.J.); wu_548@dlmu.edu.cn (S.W.)

**Keywords:** biomimetic fish scale texture, reciprocating friction pair, discontinuous interface, lubricating oil transportation

## Abstract

Focusing on the difficulty of lubrication in the scavenging port area of a cylinder liner of an actual marine two-stroke diesel engine, the transportation of interface lubricating oil was studied. In this paper, a biomimetic fish scale texture composed of fan-shaped and arc-shaped curves is designed, and the numerical simulation model is established according to this texture. Through simulation research, the variation rules of pressure distribution, interfacial velocity, and outlet volume flow rate on the biomimetic fish scale texture surface at different velocities and temperatures are obtained. Moreover, the biomimetic fish scale texture is machined on the surface of a reciprocating friction pair by laser etching, and the oil transport speed of the interface is tested under different conditions. The results show that the existence of the biomimetic fish scale texture on the friction pair can effectively improve the pressure difference between interfaces during reciprocating motion. The pressure difference enhances the flow properties of interfacial lubricating oil, thereby improving its mass transport capacity. In addition, increasing the movement speed and oil temperature can increase the oil transport speed of interfacial lubricating oil. The results of the experiment suggest that, under continuous and discontinuous interface conditions, compared with a friction pair without texture, the improvement rate of the lubricating oil transport speed at the interface of the friction pair with the biomimetic fish scale texture can reach 40.7% and 69.1%, respectively.

## 1. Introduction

Reciprocating friction pairs are an important part of marine two-stroke diesel engines. The excellent lubrication state of traditional friction pairs, such as piston–cylinder liners, directly affects the working performance of diesel engines [1,2,3]. When the lubricating oil flows through the scavenging port area of the cylinder liner, the lubricating oil transportation path will be interrupted, resulting in an oil-depleted state at the distal end of the scavenging port [4,5]. This condition will aggravate the wear at the scavenging port, but excessive oil supply will cause carbonization at the piston head, which will also destroy the lubrication of the piston ring and cylinder liner. Therefore, it is of great significance to realize methods of transportation for more lubricating oil under the condition of a certain supply of lubricating oil in friction pairs.

Many studies have shown that reasonable surface texture can greatly reduce the friction between friction pairs in a piston–cylinder liner reciprocating system to improve lubrication conditions [6,7,8]. Biologically inspired surface textures are also important research areas in piston–cylinder liner tribology [9,10,11,12,13]. Lu [14] designed a texture that can be applied to the cylinder liner–piston ring with reference to the microstructure of the bellied snake scale. The experimental results show that the combination of tooth-like structures and pits in the texture of snake belly scales can enhance the lubrication effect. Gao [15] designed and processed a variety of cylindrical pit array pistons inspired by earthworms and leeches. The friction test shows that the friction and wear performance of cylindrical pit array pistons is excellent, and the wear of some textured pistons is reduced by more than 50%. Since fish scale texture has excellent fluid drag reduction characteristics, there are many related tribological studies [16,17,18,19]. Quan [20] made numerical analyses of carp scale texture, finding that the friction coefficient of the piston surface clearly decreased after adding the fish scale texture. Chen [21] fabricated a periodic array of fish scales on a polished aluminum template through laser ablation and measured the drag reduction performance of the biomimetic fish scale surface in a circulating water tunnel, with a maximum drag reduction rate of 10.26%.

The existing research on lubricating oil transport on textured surface is mainly based on the fixed grooved surface, and the directional transport of lubricating oil is realized by the capillary action of surface tension and wettability [22,23,24,25]. Gong [26] processed texture on the turning tool, theoretically analyzed its lubricant transport mechanism, and revealed a change in surface wetting speed caused by capillary action during transport. Guo [27] developed an underwater super-oleophilic two-dimensional surface with an asymmetric oleophobic barrier by superhydrophobic spraying and laser etching, which led to asymmetric diffusion resistance, and achieved the unidirectional and long-distance transport of oil droplets. Therefore, it is of great significance to carry out research on interfacial lubricating oil transport based on moving surfaces to expand the field of oil transport.

Because the arrangement of fish scales is forward, the friction forces in different water flow directions are different, which indicates that fish scales have the characteristics of friction anisotropy [28,29,30]. Bixler and Bhushan [31] found that sinusoidal grooves on rice leaves and banded scales arranged on butterfly wings provide anisotropic flow. Wang [32] found that honey bee tongue bristles are hard and hydrophobic, but they can draw honey in through several approximate reciprocating motions. These examples provide us with new ideas for anisotropic textures in the field of fluid transport.

**Figure 1:**
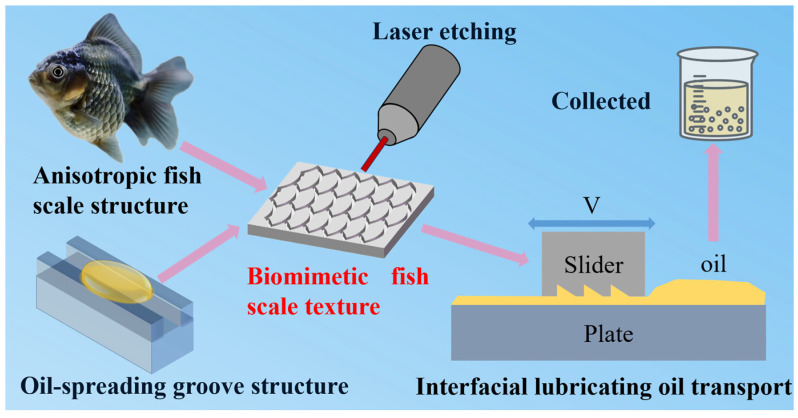
Research ideas for interfacial lubricating oil transport.

In this paper, inspired by the asymmetric structure of fish scales and the groove structure with directional fluid transport, a pit texture composed of fan-shaped and arc-shaped curves is designed. The friction pair with a bionic fish scale texture is simulated and analyzed, and the effects of wall velocity and lubricating oil temperature on the interface lubricating oil transport characteristics are studied. Then, the lubricating oil transportation speed of the biomimetic fish scale-textured piston and the non-textured piston under different working conditions is compared through experiments, and the simulation results are verified. Research ideas for interfacial lubricating oil transport are shown in Figure 1. This work provides a theoretical basis for the design of reciprocating friction pairs aimed at improving the interfacial lubricating oil transport characteristics.

## 2. Research Methods

### 2.1. Simulation Model

#### 2.1.1. Biomimetic Fish Scale Texture Design

**Figure 2:**
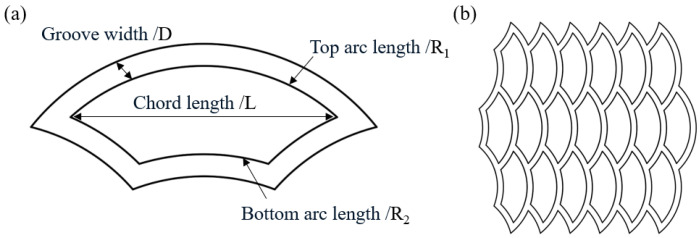
The biomimetic fish scale texture designed in this paper: (**a**) single texture and geometric parameters; (**b**) textured array.

A schematic diagram of a single and multiple biomimetic fish scale texture is shown in Figure 2. Regarding the geometric parameters, *L* is the chord length (mm), *R*_1_ is the top arc length (mm), *R*_2_ is the bottom arc length (mm), and *D* is the groove width and the width at each position is consistent.

#### 2.1.2. Numerical Simulation

**Figure 3:**
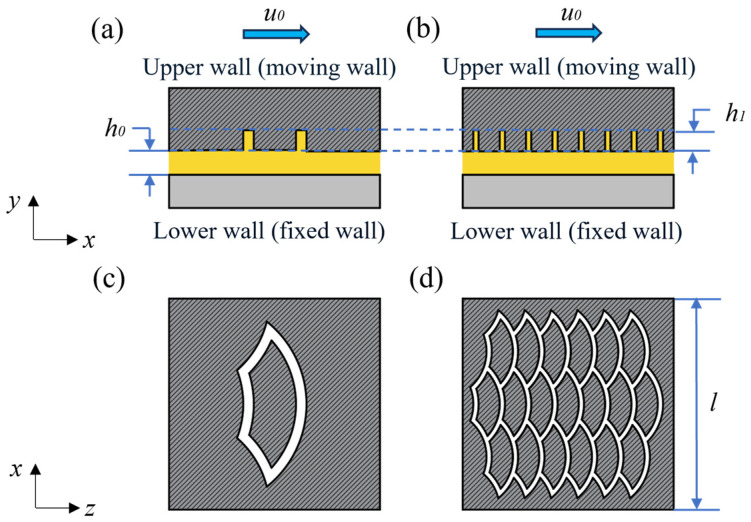
A biomimetic fish scale texture fluid model: (**a**) single textured front view; (**b**) textured array front view; (**c**) single textured top view; (**d**) textured array top view.

In order to study the law of interfacial lubricating oil transport, a biomimetic fish scale texture fluid model is established. A biomimetic fish scale texture fluid model is shown in Figure 3, where (a) and (c) are single biomimetic fish scale texture calculation units and (b) (d) are biomimetic fish scale array calculation units. The lower wall surface is fixed, while the upper wall surface is movable and has a biomimetic fish scale texture. The wall motion velocity is *u*_0_ (m/s), the side length of the fluid is *l* (mm), the minimum oil film thickness is *h*_0_ (mm), and the depth of the texture is *h*_1_ (mm).

#### 2.1.3. Fluid Control Equations

In this paper, the numerical model of the fluid flow of the biomimetic fish scale texture is established and solved according to the N-S equation. At the same time, the following assumptions are made: 1. The lubricating medium is an incompressible Newtonian fluid, regardless of the influence of volume force. 2. The fluid flow is steady, and the wall fluid velocity is the same as the wall movement velocity. 3. The surface of the friction pair does not change. 4. Some basic assumptions were made regarding the remaining N-S equations.

Based on the above assumptions, the expansion of the N-S equation in the *x*, *y*, and *z* directions is as follows:

(1) $$\rho \left(u\frac{\partial u}{\partial x}+v\frac{\partial u}{\partial y}+w\frac{\partial u}{\partial z}\right)=-\frac{\partial p}{\partial x}+\eta \left(\frac{\partial^{2}u}{\partial x^{2}}+\frac{\partial^{2}u}{\partial y^{2}}+\frac{\partial^{2}u}{\partial z^{2}}\right)$$

(2) $$\rho \left(u\frac{\partial v}{\partial x}+v\frac{\partial v}{\partial y}+w\frac{\partial v}{\partial z}\right)=-\frac{\partial p}{\partial y}+\eta \left(\frac{\partial^{2}v}{\partial x^{2}}+\frac{\partial^{2}v}{\partial y^{2}}+\frac{\partial^{2}v}{\partial z^{2}}\right)$$

(3) $$\rho \left(u\frac{\partial w}{\partial x}+v\frac{\partial w}{\partial y}+w\frac{\partial w}{\partial z}\right)=-\frac{\partial p}{\partial z}+\eta \left(\frac{\partial^{2}w}{\partial x^{2}}+\frac{\partial^{2}w}{\partial y^{2}}+\frac{\partial^{2}w}{\partial z^{2}}\right)$$

Continuity equation:

(4) $$\frac{\partial u}{\partial x}+\frac{\partial v}{\partial y}+\frac{\partial w}{\partial z}=0$$

where ρ is the fluid density (g/m^3^), *u*, *v*, and *w* are the flow rates in the *x*, *y*, and *z* directions (m/s), p is the average pressure (kPa), and η is the lubricating oil viscosity (mm^2^/s).

#### 2.1.4. Forward and Reverse Motion Regulations

**Figure 4:**
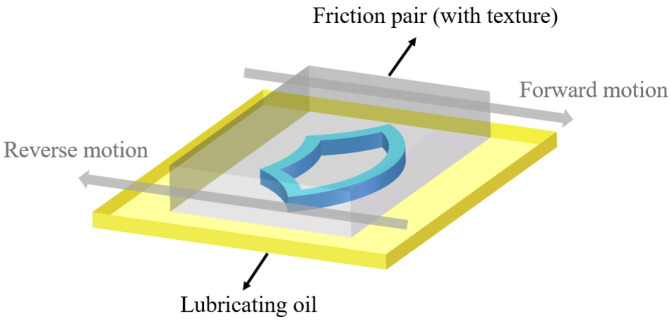
Schematic diagram of forward and reverse movement of biomimetic fish scale texture friction pair.

Because the lubricating oil flows through the top arc and bottom arc of the biomimetic fish scale texture in the opposite direction of motion, the sequence is different. A schematic diagram of the forward and reverse movement of the biomimetic fish scale texture friction pair is shown in Figure 4.

#### 2.1.5. Solution Procedure

**Figure 5:**
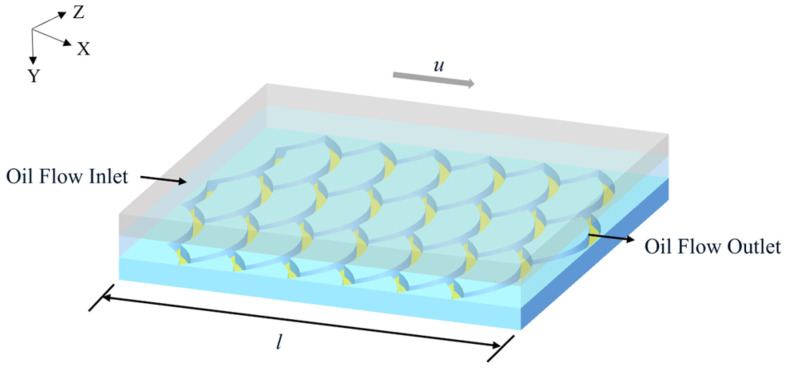
The biomimetic fish scale texture simulation calculation domain.

In this paper, the three-dimensional double-precision solver is used for the simulation calculation in Fluent software 2024 R2, the volume of the fluid multiphase flow model is adopted, and the two phases are oil and air, opening up surface tension model and wall adhesion options. The pressure–velocity coupling is solved using the Coupled scheme, with the QUICK discretization scheme applied to both the momentum and energy equations. The PRESTO! scheme is adopted for pressure interpolation. A convergence criterion of 10^−5^ was specified for all governing equations, while other computational parameters maintained their default settings to streamline the numerical process. In order to facilitate the calculation, the remaining conditions are chosen as the default. The biomimetic fish scale texture simulation calculation domain is shown in Figure 5. The inlet is set as an oil flow inlet, the outlet is set as an oil flow outlet, and the center surface in the groove and the interface is set as a monitoring surface. After referring to the selection of texture parameters in the relevant papers [16,26], we finally design the texture parameters shown in Table 1.

### 2.2. Experiment Preparation

#### 2.2.1. Experiment Sample

Consistent with the actual working conditions, the piston sample is processed from the aluminum alloy material. To avoid the influence of the oil scraping effect of the piston ring on the measurement of interface lubricating oil, a special piston without a piston ring or ring groove is used in this test. In order to ensure smooth movement, this experiment adopts a piston group with upper and lower pistons connected.

In this study, the biomimetic fish scale texture is processed on the surface of piston samples by the laser etching method. The laser processing equipment adopts the SHGX-20 laser processing system (Shenyang, China). Its maximum power is 20 W, its wavelength is 1064 nm, and its minimum line width is 0.012 mm. The biomimetic fish scale texture is drawn using the drawing software SHANHE 2.14.16, which comes with the laser marking machine, and the processing depth is controlled by changing the laser processing power, number of laser scans, and laser scanning speed. The laser processing parameters of the biomimetic fish scale texture are shown in Table 2.

**Figure 6:**
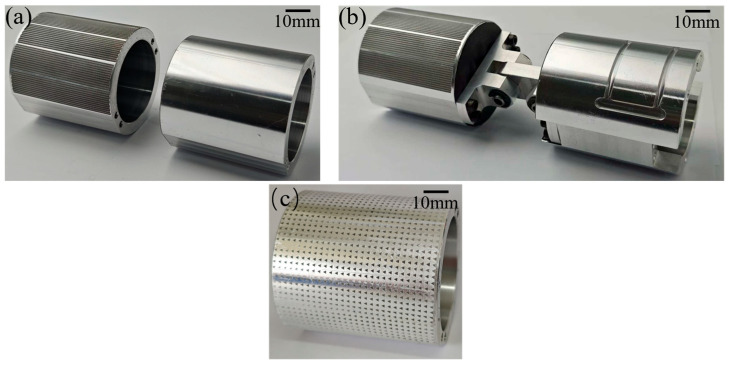
The physical drawings of pistons: (**a**) pistons without texture and those processed with a biomimetic fish scale texture; (**b**) piston group; (**c**) pistons processed with a triangular texture.

The following steps included putting the laser-processed textured piston into an ultrasonic cleaning machine, washing away the processing residue with alcohol, and drying to obtain the prepared textured piston. Pistons without texture and those processed with a biomimetic fish scale texture are shown in Figure 6a. A piston group mounted with an upper piston with the biomimetic fish scale texture is shown in Figure 6b. In order to prove that the biomimetic fish scale texture demonstrates excellent interfacial lubricating oil transport performance, a common asymmetrically textured triangle [33] is also processed and compared with it, which is shown in Figure 6c. The parameters of these pistons are shown in Table 3.

**Figure 7:**
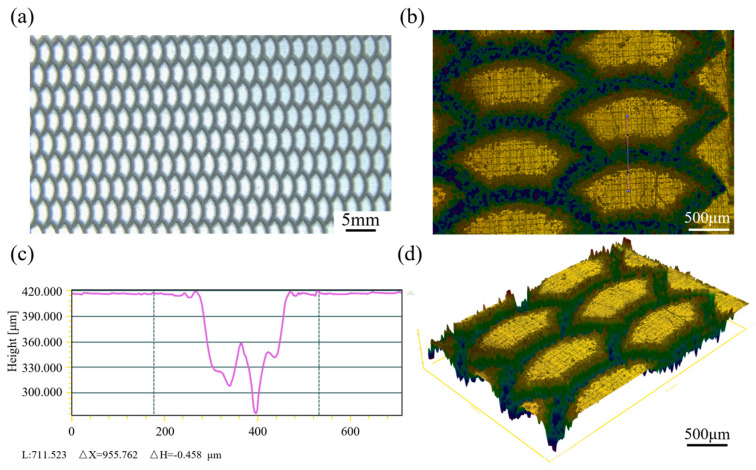
Pistons with a biomimetic fish scale texture: (**a**) macro diagram; (**b**) two-dimensional white light interferogram; (**c**) processing depth curve diagram; and (**d**) three-dimensional white light interferogram.

The observation of the texture was carried out using a stereo microscope (Nikon SMZ745T, Saitama, Japan), and the macro image is shown in Figure 7a. Then, a white light interference lens in MFT-5000 (Rtec, San Jose, CA, USA) was used to observe the surface topography, and the Lambda topography system was used to generate two-dimensional and three-dimensional dimensional white light interference topography maps (Figure 7b,d), and the processing depth curve diagram (Figure 7c) is generated by underlining in Figure 7b.

**Figure 8:**
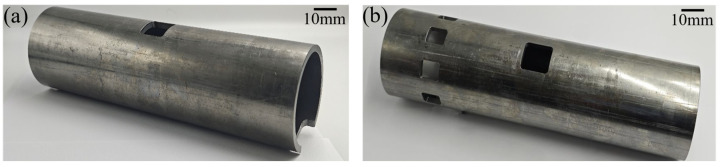
The physical drawings of cylinder liners: (**a**) continuous interface condition; (**b**) discontinuous interface condition.

Considering that, in the actual piston–cylinder liner system, there is a scavenging port on the cylinder liner, this can be considered a discontinuous interface. To explore whether the biomimetic fish scale texture can enhance the lubricating oil delivery capacity in the discontinuous interface so that more lubricating oil can cross the scavenging port and realize the distal lubrication, in this experiment, two kinds of cylinder liners with continuous and discontinuous surfaces were designed and machined to study the lubricating oil transport under continuous and discontinuous interface conditions, respectively. The physical drawings of cylinder liners are shown in Figure 8, and the material used is high-quality carbon steel. The parameters of the cylinder liners are shown in Table 4.

#### 2.2.2. Test System

**Figure 9:**
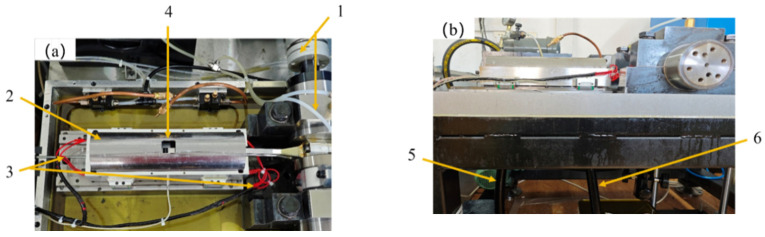
Interfacial lubricating oil transport test system: (**a**) Top view (**b**) Front view. 1. crankshaft and crankshaft lubrication system; 2. piston and cylinder liner; 3. cylinder liner heating system; 4. oil supply port; 5. oil pipe (measurement); 6. oil pipe (recovery).

The interfacial lubricating oil transport test system based on the reciprocating friction pair is shown in Figure 9. It is mainly composed of a crankshaft lubrication system, reciprocating mechanism, oil supply system, cylinder liner heating system, and control system. This test system is mainly used to test the transport speed of the interfacial lubricating oil of the reciprocating friction pair so as to evaluate the transport enhancement effect that the biomimetic fish scale texture exerts.

**Figure 10:**
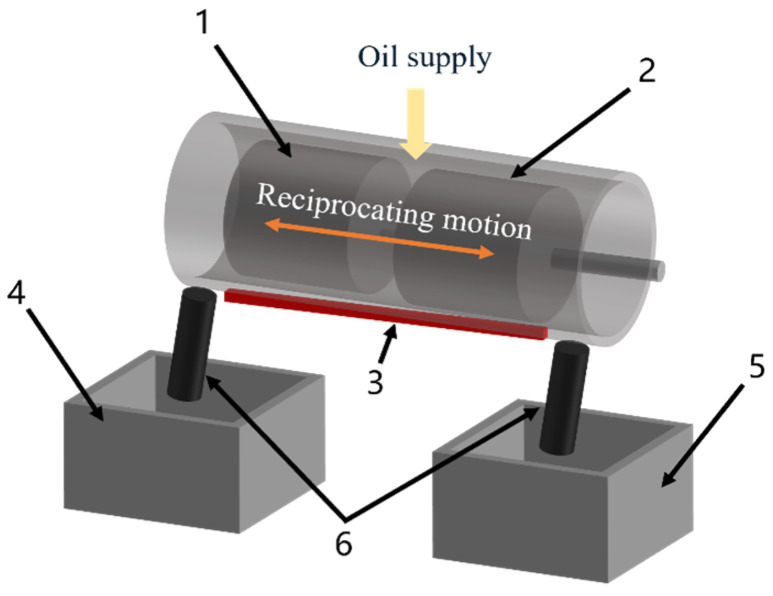
Interfacial lubricating oil transport speed test schematic diagram: 1. upper piston (two types); 2. lower piston; 3. cylinder liner heating device; 4. oil collection device (weighing); 5. oil collection device (recovery); 6. oil pipeline.

The interfacial lubricating oil transport speed test schematic diagram is shown in Figure 10. An upper piston without texture and an upper piston with the biomimetic fish scale texture are installed at the left end. Then, the opening of the cylinder liner is filled with the oil, and the oil is heated to the required temperature by the heating equipment in advance. Meanwhile, the testing machine is turned on to perform the reciprocating motion of the piston group. After the test, the left-end oil collection device is weighed, and the oil transport speed is calculated.

The formulas for the transport speed, *Q*, and transport speed increase rate, *η*, are as follows:

(5) $$Q=\frac{M_{1}-M_{0}}{T}\times 100\%$$

(6) $$\eta =\frac{Q_{1}-Q_{0}}{Q_{0}}\times 100\%$$

In the formula, *M*_0_ (g) is the mass of the oil collection device before the transportation test and *M*_1_ (g) is the mass of the oil collection device after the transport test. *T* (min) is the time at which the oil transport test was performed. *Q*_0_ (g/min) is the oil transport speed of the untextured upper piston. *Q*_1_ (g/min) is the oil transport speed of the upper piston with the biomimetic fish scale texture.

In this experiment, by changing the parameters of the oil temperature, crankshaft speed, and oil supply rate, the oil transport speed of two kinds of pistons under different working conditions was compared. Then, the increase rate of the oil transport speed is calculated, and the correctness and effectiveness of the simulation results are verified by the experimental results.

## 3. Results and Discussion

### 3.1. Simulation Results

The parameters in Table 1 are substituted into the simulation model, and the variation laws of the surface pressure distribution, interface flow velocity, and outlet volume flow rate of the biomimetic fish scale texture at different velocities and temperatures are analyzed.

**Figure 11:**
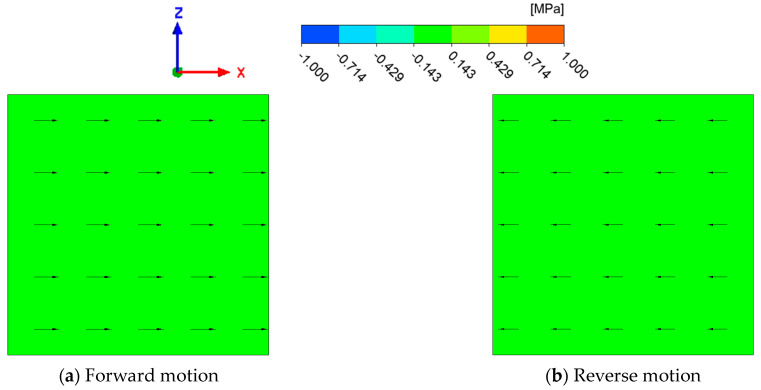
Interfacial pressure distribution of untextured surface (Arrows are velocity vector).

In order to confirm the feasibility of the simulation method, firstly, the untextured surface is simulated at a lubricating oil temperature of 30 °C and a moving speed of 5 m/s. The simulation results are shown in Figure 11. It can be seen that the interface pressure of the untextured surface is basically the same under the forward and reverse movement, and there is no high-pressure area or low-pressure area, which means the flow of the interface lubricating oil cannot be improved.

#### 3.1.1. Effect of Wall Velocity on Interface Oil Transport Using Biomimetic Fish Scale Texture

In this part, under the condition of an oil temperature of 30 °C, the influence of velocity on lubricating oil transport at the biomimetic fish scale texture interface is studied by changing the wall movement velocity.

**Figure 12:**
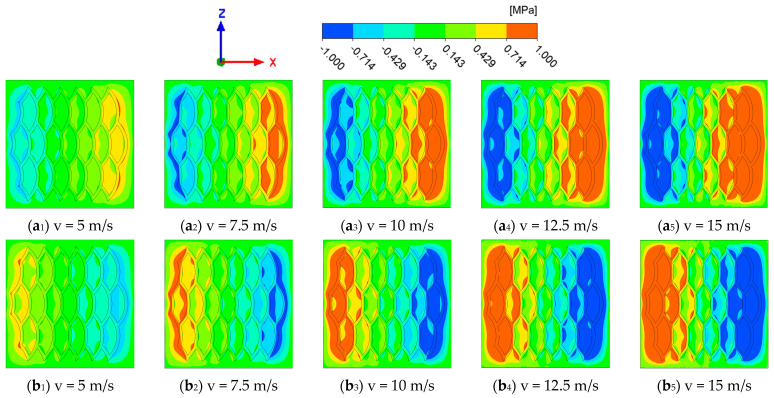
Interfacial pressure distribution of biomimetic fish scale texture at different velocities: (**a**) forward motion; (**b**) reverse motion.

Figure 12 shows the change in pressure distribution at the interface of the biomimetic fish scale texture with the increase in the wall motion speed. In forward motion, with the increase in wall motion, the area of the outlet high-pressure area and inlet low-pressure area gradually increases. In reverse motion, the pressure field shows the same tendency. This situation leads to an increase in the pressure difference generated by the forward and reverse motion, which promotes the movement of fluid between interfaces.

**Figure 13:**
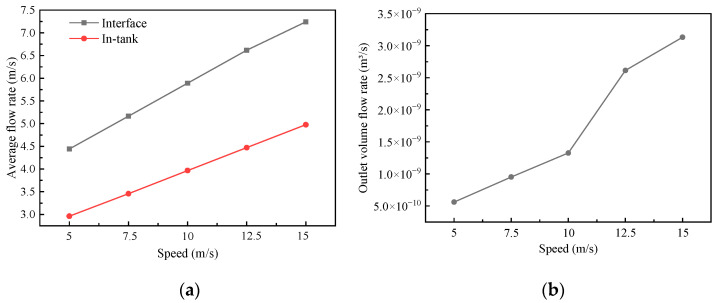
The variation in the transport characteristics parameters of the biomimetic fish scale texture with the wall movement velocity: (**a**) average flow rate; (**b**) volume flow rate.

Figure 13a shows the change in the oil flow rate with the wall movement velocity. With the increase in wall velocity, the flow of lubricating oil between interfaces and in the groove intensifies, and the rate between interfaces clearly increases. Figure 13b shows the change in the volume flow rate at the oil outlet with the wall motion velocity. The volume flow rate also increases with the increase in the wall motion velocity, which indicates that the transport of interfacial lubricating oil is enhanced.

#### 3.1.2. Effect of Oil Temperature on Interface Oil Transport at Biomimetic Fish Scale Texture

In this part, under the condition of a wall movement velocity of 5 m/s, the influence of velocity on oil transport at the biomimetic fish scale texture interface is studied by changing the oil temperature.

**Figure 14:**
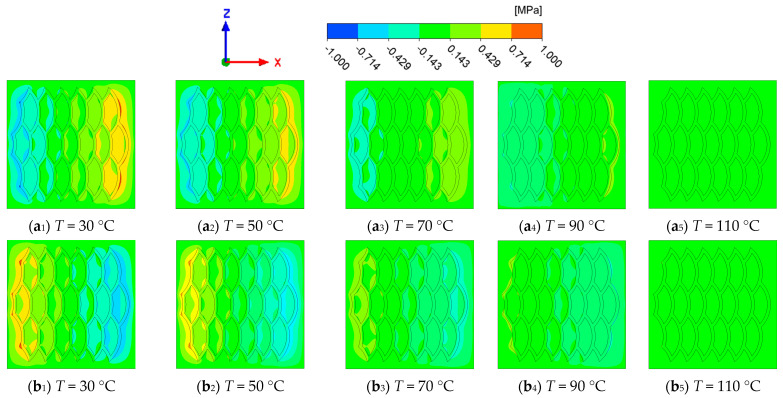
Interfacial pressure distribution of biomimetic fish scale texture at different oil temperature: (**a**) forward motion; (**b**) reverse motion.

Figure 14 shows the change in pressure distribution at the interface of the biomimetic fish scale texture with the increase in oil temperature. With the increase in oil temperature, the viscosity of lubricating oil decreases, which makes the area of the high-pressure zone and low-pressure zone decrease, but the influence of the average interface pressure is small.

**Figure 15:**
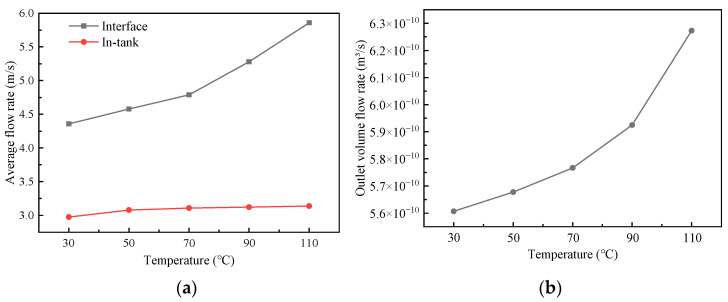
The variation in the transport characteristic parameters of the biomimetic fish scale texture with oil temperature: (**a**) average flow rate; (**b**) volume flow rate.

Figure 15a shows the change in the oil flow rate with the oil temperature. With the increase in oil temperature, the viscosity of lubricating oil decreases, which makes the average rate of lubricating oil between interfaces increase. The flow in the groove is affected by the decrease in viscous stress and shows little change. Figure 15b shows the change in the volume flow rate at the oil outlet with the oil temperature. The volume flow rate also increases with the increase in oil temperature, which indicates that the transport of interfacial lubricating oil is enhanced.

### 3.2. Experimental Verification

#### 3.2.1. Effect of Piston Velocity and Oil Supply Rate on Lubricating Oil Transport Characteristics Under Continuous Interface Condition

Using the test system of the interfacial lubricating oil transport of the reciprocating friction pair, the characteristics of interfacial lubricating oil transport are studied. This experiment was carried out in a continuous cylinder liner with the lubricating oil temperature maintained at 25 °C. The simulation is verified by comparing the transport speeds of the untextured piston and the biomimetic fish scale-textured piston at different crankshaft speeds and oil supply rates. In addition, the experiment of the triangular piston is added. Detailed test parameters are shown in Table 5.

**Figure 16:**
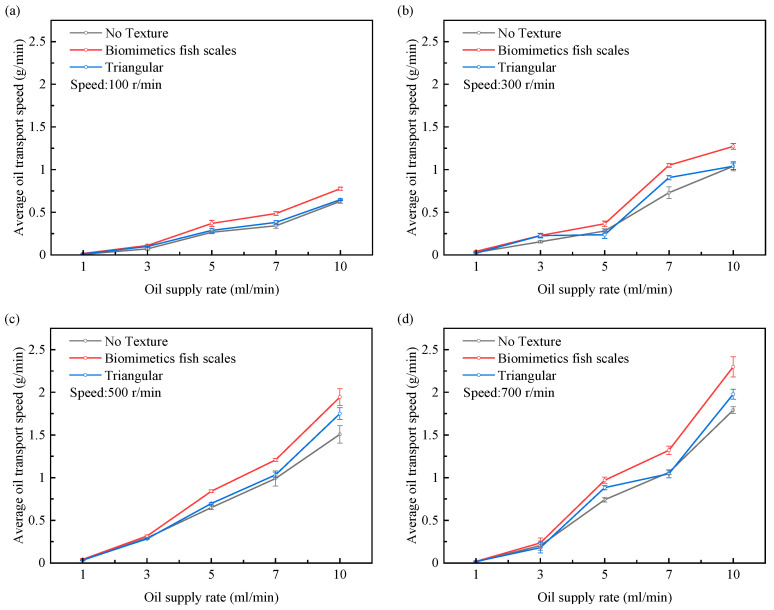
Effects of oil supply rate and crankshaft speed on lubricating oil transport speed at continuous interface. (**a**) speed = 100 r/min; (**b**) speed = 300 r/min; (**c**) speed = 500 r/min; (**d**) speed = 700 r/min.

As shown in Figure 16, with the increase in the crankshaft speed, the transport speed of various surface pistons increases, but the lifting rate of the biomimetic fish scale texture piston is the highest. The simulation results show that the pressure difference between the interfaces of the biomimetic fish scale texture friction pair increases with the increase in velocity during reciprocating motion. The increase in the pressure difference between interfaces promotes the flow of lubricating oil. With the increase in the oil supply rate, the lubrication conditions gradually improve, and the transport speed of the two pistons also increases. It can be concluded that increasing the crankshaft speed and oil supply can improve the transportation capacity of the piston-lubricating oil. Compared with the non-textured piston and the piston with a triangular texture, the lubricating oil transport speed of the piston with the biomimetic fish scale texture is faster under various working conditions.

#### 3.2.2. Effect of Oil Temperature on Lubricating Oil Transport Characteristics Under Continuous Interface Condition

In this experiment, the oil supply rate is 10 mL/min, the crankshaft speed is 700 r/min, and the oil supply time is 10 min. By changing the lubricating oil temperature (25–110 °C), the transport speed of the non-textured piston and the piston with the biomimetic fish scale texture was compared.

**Figure 17:**
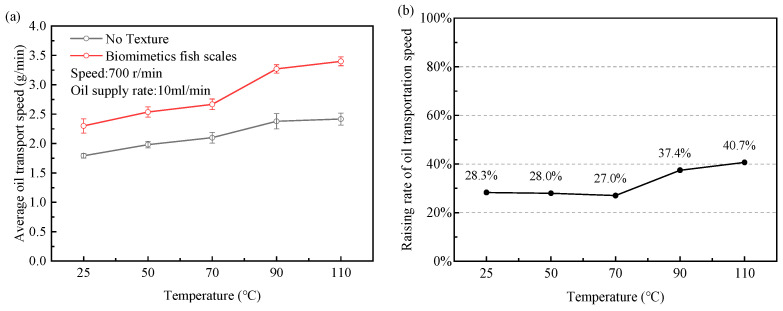
Effects of oil temperature on lubricating oil transport speed at continuous interface: (**a**) lubricating oil transport speed; (**b**) increase rate of oil transport speed of biomimetic fish scale-textured pistons compared to non-textured pistons.

As shown in Figure 17a, increasing the lubricating oil temperature can further increase the transport speed of lubricating oil. The simulation results show that the flow resistance of lubricating oil decreases when the temperature increases and the average speed of lubricating oil between interfaces increases. Pistons with a biomimetic fish scale texture transport faster relative to non-textured pistons. As shown in Figure 17b, when the temperature increases, the lift rate of the transport speed also increases. Under the working conditions of a speed of 700 r/min, temperature of 110 °C, oil supply rate of 10 mL/min, and oil supply time of 10 min, the transportation speed of the biomimetic fish scale-textured piston is increased by 40.7% compared with that of the non-textured piston.

#### 3.2.3. Effect of Piston Velocity and Oil Supply Rate on Lubricating Oil Transport Characteristics Under Discontinuous Interface Condition

This experiment was carried out in a continuous cylinder liner with the lubricating oil temperature maintained at 25 °C. By comparing the transport speeds of the non-textured piston and the biomimetic fish scale-textured piston at different crankshaft speeds and oil supply rates, the possibility of lubricating oil transport under discontinuous interface conditions is explored. Detailed test parameters are shown in Table 6.

**Figure 18:**
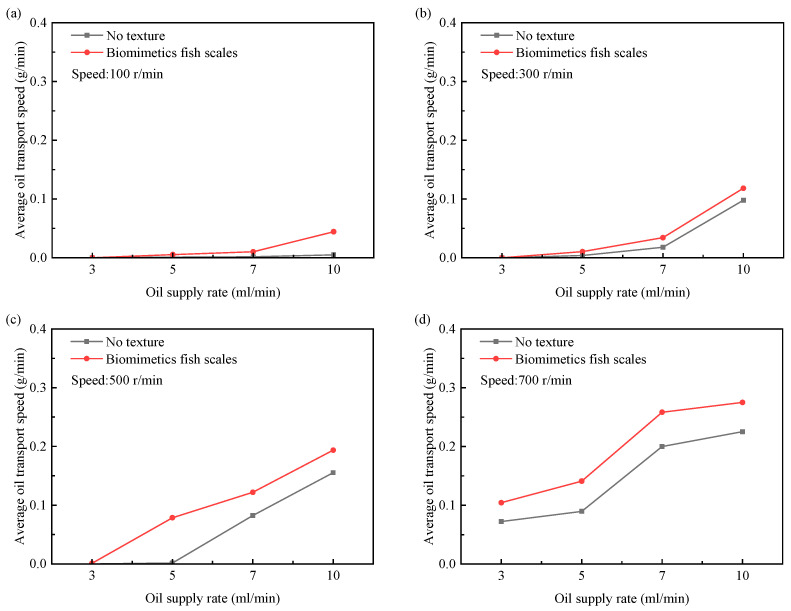
Effects of oil supply rate and crankshaft speed on lubricating oil transport speed at discontinuous interface. (**a**) speed = 100 r/min; (**b**) speed = 300 r/min; (**c**) speed = 500 r/min; (**d**) speed = 700 r/min.

As shown in Figure 18, when the crankshaft speed is low, even under the condition of a high oil supply rate, most of the lubricating oil flows out with the scavenging port and the lubricating oil transport speed of both pistons is very low. With the increase in the crankshaft speed, more lubricating oil passes over the scavenging port, and the oil transportation speed further increases. The oil transport speed reaches the highest at high oil supply rates. Compared with non-textured pistons, pistons with a biomimetic fish scale texture require lower crankshaft speeds and oil supply rates to realize lubricating oil transportation under various working conditions.

#### 3.2.4. Effect of Oil Temperature on Lubricating Oil Transport Characteristics Under Discontinuous Interface Condition

In this experiment, the oil supply rate is 5 mL/min, the crankshaft speed is 700 r/min, and the oil supply time is 5 min. By changing the lubricating oil temperature (25–90 °C), the transport speed of the non-textured piston and the piston with a biomimetic fish scale texture is compared.

**Figure 19:**
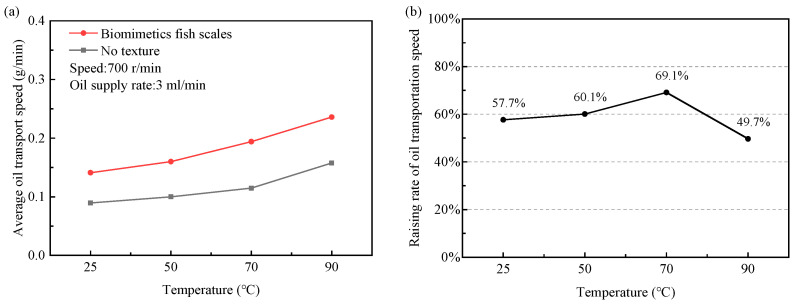
Effects of oil temperature on lubricating oil transport speed at discontinuous interface: (**a**) lubricating oil transport speed; (**b**) increase rate of oil transport speed of biomimetic fish scale textured pistons compared to non-textured pistons.

As shown in Figure 19a, increasing the lubricating oil temperature can further increase the transport speed of lubricating oil. Pistons with a biomimetic fish scale texture transport faster relative to non-textured pistons. As shown in Figure 19b, under the working conditions of a speed of 700 r/min, temperature of 70 °C, oil supply rate of 5 mL/min, and oil supply time of 5 min, the transportation speed of the biomimetic fish scale-textured piston is increased by 69.1% compared with that of the non-textured piston. When the temperature is increased to 90 °C, the improvement rate of transport speed decreases. This is because the viscosity of the lubricating oil is significantly reduced and the fluidity of the oil is enhanced in the temperature range of 70–90 °C, so that it is easier for the oil to flow out of the scavenging port. Even in this case, the piston with a biomimetic fish scale texture still shows superior interfacial lubricating oil transport ability.

## 4. Conclusions

This research employs a combination of simulation and experiments to investigate the effect of a biomimetic fish scale texture on lubricating oil transport at reciprocating friction pair interfaces. Through numerical simulation parameters, such as the pressure distribution and interface rate, the effects of movement velocity and temperature on the interfacial lubricating oil transport characteristics of the friction pair with a biomimetic fish scale texture are analyzed. Through experiments, the transport speed of the friction pairs with a biomimetic fish scale texture under various working conditions is obtained and the accuracy of the numerical simulation is verified.

(1) The presence of the biomimetic fish scale texture on the friction pair can effectively improve the pressure difference between interfaces during reciprocating motion. This will enhance the flow properties of interfacial lubricating oil, thus improving the transport capacity of interfacial lubricating oil.

(2) When the wall velocity is increased, the pressure difference between interfaces will be further increased and the flow velocity between interfaces will increase. This leads to an increase in the interface flow rate and an increase in the outlet volume flow rate. It can be concluded that increasing the wall movement velocity is beneficial to interfacial lubricating oil transport. When the lubricating oil temperature is increased, the viscosity of the lubricating oil decreases and the flow rate between interfaces increases. This leads to an increase in the interface flow rate and an increase in the outlet volume flow rate. It can be concluded that increasing the oil temperature is beneficial to the interfacial lubricating oil transport.

(3) For both continuous and discontinuous interface conditions, under various working conditions, the lubricating oil transport speed at the interface of the friction pairs with the biomimetic fish scale texture is higher than that of friction pairs without texture.

For continuous interface conditions, the optimal oil transport speed improvement rate is 40.7% under the working conditions of a rotational speed of 700 r/min, temperature of 110 °C, oil supply rate of 10 mL/min, and oil supply time of 10 min. For discontinuous interface conditions, the optimal oil transport speed improvement rate is 69.1% under the working conditions of a rotational speed of 700 r/min, temperature of 70 °C, oil supply rate of 5 mL/min, and oil supply time of 5 min.

(4) In this study, an attempt was made to directly compare the volume flow rate in the simulation results with the transport speed in the experimental results after unification. In this simulation study, only the difference between the forward and reverse motions of the biomimetic fish scale-textured friction pair is considered, and the unsteady simulation based on reciprocating motion (or sinusoidal motion) is not carried out, so it cannot be compared with the experimental results of the actual reciprocating friction pair at variable speeds. This leads to around a tenfold numerical difference between the volume flow rate obtained in the simulation and the transport speed obtained in the experiment after the unit is unified. However, the volume flow rate in the simulation is consistent with the transport speed in the experiment, which can also prove the accuracy of the simulation.

The results of this study demonstrate the potential of a fish scale texture in enhancing the lubricating properties of the distal end of reciprocating friction pairs. It will provide valuable insights for future investigations and practical applications in oil transportation, as well as providing inspiration for designs for medical, marine, and industrial applications.

## Figures and Tables

**Table 1:** Parameters of the biomimetic fish scale texture model.

Parameter Names		Numerical
Texture parameters	Chord length *L* (mm)	1.2
	Groove width *D* (mm)	0.1
	Top arc length *R*_1_ (mm)	1.32
	Bottom arc length *R*_2_ (mm)	0.59
	Fluid domain side length *l* (mm)	4 × 4
	Texture depth *h*_1_ (mm)	0.1
Fluid domain parameters	Initial oil film thickness *h*_0_ (mm)	0.1
	Viscosity of lubricating oil *η* (Pa·s)	0.169
	Density of lubricating oil *ρ* (kg·m^−3^)	868
	Atmospheric reference pressure *p*_0_ (Pa)	101,325.1

**Table 2:** Laser processing parameters of biomimetic fish scale texture.

Laser Processing Parameter	Numerical
Laser processing power (W)	20
Laser scanning speed (mm/s)	100
Number of laser scans	5
Single processing area (mm × mm)	65 × 15
Number of laser etching of a single piston	10

**Table 3:** Parameters of pistons.

Parameters of Piston		Numerical
Piston parameters	Piston length (mm)	67
	Piston outer diameter (mm)	65
	Piston wall thickness (mm)	7.5
Characteristic parameters of biomimetic fish scale-textured piston	Chord length *L* (mm)	1.2
	Groove width *D* (mm)	0.1
	Top arc length *R*_1_ (mm)	1.32
	Bottom arc length *R*_2_ (mm)	0.59
	Texture depth (mm)	0.1
Characteristic parameters of triangular-textured piston	Textured side length (mm)	0.8
	Texture spacing (mm)	0.8
	Texture depth (mm)	0.1

**Table 4:** Parameters of cylinder liners.

Parameters of Cylinder Liners		Numerical
Continuous interface condition	Cylinder liner length (mm)	278
	Cylinder liner outer diameter (mm)	70
	Cylinder liner wall thickness (mm)	5
Discontinuous interface condition	Cylinder liner length (mm)	278
	Cylinder liner outer diameter (mm)	70
	Cylinder liner wall thickness (mm)	5
	Distance between scavenging port and upper end (mm)	37.5
	Scavenging port length (mm)	17.5
	Scavenging port width (mm)	15
	Number of scavenging ports	8

**Table 5:** Parameters of test of lubricating oil transport characteristics under continuous interface condition.

Parameter	Numerical
Lubricating oil temperature	25 °C
Oil supply rate	1 mL/min, 3 mL/min, 5 mL/min, 7 mL/min, 10 mL/min
Crankshaft speed	100 r/min, 300 r/min, 500 r/min, 700 r/min
Oil supply time	10 min

**Table 6:** Parameters of test of lubricating oil transport characteristics under discontinuous interface condition.

Parameter	Numerical
Lubricating oil temperature	25 °C
Oil supply rate	3 mL/min, 5 mL/min, 7 mL/min, 10 mL/min
Crankshaft speed	100 r/min, 300 r/min, 500 r/min, 700 r/min
Oil supply time	5 min

## Data Availability

The original contributions presented in this study are included in the article. Further inquiries can be directed to the corresponding author.
